# Editorial Chit-Chat

**Published:** 1878-07

**Authors:** 


### Editorial Chit-Chat.

FOURTH OF JULY—and fire crackers only eight cents a pack! Spts-spts-bang!

A LITTLE LATE.—Our July number is always a week late by reason of our vacation.

THE SCORE.— The thermometer has made 120 out of a possible 150, but promises to do better in a day or two.

AUSBURN TOWNER. — Our contributor, Mr. Towner, has a responsible position upon the editorial staff of the New York *Sun*, and that journal already exhibits evidences of the presence of his skillful pen.

WHICH?—Either the completion of the academic course has beautified the young ladies of the graduating class, not only mentally but personally, as well; else were all the prettiest girls selected to graduate at the Academy in the class of '78.

OUR SCHOOLS—Elmira is justly proud of her grammar schools and Academy. We are glad to see the commencement exercises of all, well attended, the buildings were unable to hold the people who applied for admission. It is a good sign when parents are proud of the achievements of their children at school—it argues well for the system and the teachers.

BOUND BISTOURYS.—We have had the BISTOURY nicely bound and fully indexed, so that it is quite an encyclopédia of information, and can send it, post-paid, on receipt of $2.50.

It contains all the numbers from April, 1875, to April, 1878, inclusive.

OUR VACATION.—By reason of the rainy season, our trip to the woods was cut short a week. We had a good time, however, while we did remain, and do not count the expedition a failure by any means. In another article we have taken pains to note the preparations necessary for a camping excursion to the woods, that is inexpensive, yet complete in all its details. No better vacation can be taken, or spent in a more healthful and delightful manner, than living in a tent in the woods, upon some pleasant stream or lake, for a month or so, during the hot season, now approaching.

THE PARAGRAPHERS.—Both our dailys—the *Advertiser* and *Gazette*, sparkle with the wit and wisdom of the paragraphers. We need to have our news in the smallest number of words this hot weather, and the gentlemen referred to are doing their work handsomely.

SEEDS.—Green informs us that he has sold seeds and plants all over the United States, from his advertisement in this journal. Glad to hear it. We hope our patrons will continue their patronage, as he is worthy of it, and does that which he agrees to.

OUR BOYS AND GIRLS.—We were struck with the character of the declamations made by the boys and girls at the recent school commencements. The boys were all for war, the sword, statesmanship, and the like, while the girls' selections were of the blood curdling variety. They seemed to take especial delight in reciting the harrowing incidents of floods, and fires as well as startling accidents of various kinds.

A THREATENED DIVORCE.—A lean friend, possessing a fat wife, came to us this morning with a troubled face, and remarked:

"Doctor, I've been grossly insulted by my wife, and I believe I have sufficient cause to sue for a divorce."

"Why so?"

"You see how poor and lean I am? Well, this morning she told a party of ladies in my presence, that she would as soon hug a bag of clothespins as me!"

RESPONSES.—It will be seen in our correspondence that, according to request, we have heard from the horseback riders. They all seem to have entered into the affair with considerable spirit. So fascinating is this recreation, that beginners are apt to ride too far at first, then, becoming sore and tired, abandon further attempts.

Ride but a mile the first time, then gradually increase the distance daily, and you will have no trouble, but perfect enjoyment ever afterward.

A TOTAL WRECK.—Fritz rushed into his mother's presence to-day—it being the last day of school—with a triangular piece of slate, three leaves from a first reader, and the back cover of his spelling book, which he thrust into her hands, saying:

"Look 'em over, ma, and see if my books are all there!" Then, throwing his hat into the air, and, without waiting to see where it would alight, turned a summersault over the fence, and disappeared up a neighboring cherry tree.

---

ROOSTING.—Fritz was receiving a lesson upon the manner in which birds alight upon trees. He was told that pigeons, larks, robins, and blue birds always sit across t~e limb, but that all woo peckers, sit lengthwise. The conformation of the feet was explained and he seemed quite interested. Just then, he looked up and caught a glimpse of the picture of an angel, with outstretched wings, when this was his query:

"Well, which way does angels light?"

---

JUNE.—What a contemptable ninny June did make of herself, to be sure! Not only was she cold and frosty, dwarfing some flowers and completely annihilating others, but rained, drizzled and hailed us almost to the verge of distraction.

Our plants, that last year at this time were in full flower, look as though they had been showered with hot water, or had lain out a portion of the winter. But at this writing—July 1st—they seem to be reviving, giving promise of doing better, later in the season. We do hope that gentle, charming June will conduct herself in a more becoming manner next season—else will we sing her praises no more.

---

A QUEER MACHINE—is that cow-milking apparatus. We interviewed a cow to-day, who had one applied. She presented a comical appearance, while the machine was working. As the milk flowed into the pail, so relieving her well filled bag, she looked around to see where the chap was located who was doing the work. She turned her head first on the side where he naturally belonged, then toward the other. She closed her eyes a moment, as if in deep study, then suddenly looked between her fore legs, then over her back, and not seeing any one near, looked at the milk running into the pail, again seemed surprised, lifted her tail over her back, when, with a snort and a bellow, kicked pail and milking machine into atoms. Turning around, she examined what was left of the concern, smelled of it, looked at it with one eye, while she kept the other on the fellow who was gathering it up, then looking at her bag to be sure it was all there, trotted off to join her companions, with a look of withering scorn upon her otherwise amiable features, that seemed to say, none of your new-fangled milking machines for me—if you please!

WILL C. POTTER.—Mr. Towner has written a charming sketch of the late Will Potter, as he was familiarly known. Potter was a man of rare genius, and as a conversationalist was the most fascinating man we ever encountered. As Mr. Towner writes, it was quite as entertaining to hear Potter tell what he had seen in his frequent jaunts to our hills and valleys, as to have visited them yourself. His long sickness—confining him to the house for seven years, was not idly spent, for he read incessantly, and was more familiar with the best literature of his day, than most educated men. As an artist he left the evidence of his skill in many parlors of our city, showing a genius rare in one so young. Poor Potter had a hard struggle of it while he lived, yet was he as cheery and happy among his companions, as a child. He has been dead many years, but we, in common with very many of his friends and admirers, remember his many good qualities and mourn his departure from among us.

---

MEAN MEN.—Strange as it may seem, we now and then encounter a mean man—a man so mean that he is aware of his situation himself, and passes you with a hang-dog look, betokening the evil spirit that is in him. He never smiles—never manifests any pleasure in the merriment of children who chance to be playing near him—and if a dog crosses his path and ventures to wag his tail in token of recognition, a kick sends him howling from his presence. At his approach, the timid little kitten, that has been happy with the baby during the day, scampers for the window and watches for his going from a neighbor's fence. The little sparrows, that visit his back door, to pick the crumbs that fall from the table cloth, fly to the tops of the trees and chatter their displeasure—for they too have reason to fear the mean man. Neighboring children place their fingers in their mouths, and describe large orbits about him, as they quietly seek to escape to their homes. Babies cry for their pet rabbits or kittens that come to them no more, having fallen victims to the mean man's cruelty.

Have you ever met such a man? If so, shun him as you would the devil.

---

THAT WOODCHUCK.—Our greyhaired friend on the hillside, who is so expert with the rifle, and who can knock the eye and skedaddling qualities out of a "chuck" at two hundred yards, is urgently reqnested to come and try his skill on the abominable little varmint chained in our back yard.

We captured him upon our recent camping excursion, caught him in a steel trap—took him in because of the glowing accounts Squire Bodine gave us of the cute pranks *his* tame chuck did for his amusement and edification. Well, we caught the little scamp by his left fore paw, hand, or scratcher, caught him in his own home, right under the somewhat artistic mound he had erected at the rear of our tent. When he put his hand in that trap, just to see whether it would go off, it *did*, and made him squeal like fury.

We felt sorry for him, and tried our level best to free him from his painful situation. But we had to accomplish it with a long forked stick about his neck, and a pair of tongs over his snoot. What a little tiger he is, and how he did bite, scratch, squeal and throw dirt in our eyes. Finally, we got him into a box, and we came near getting into another before we got him out again. But there he is, chained to a post in a pen, to keep the children out of reach of him. We try to poke clover to him with a ten foot pole, and squirt his drink through the garden hose. If you go within gun shot of him, he shows his fine and well developed front teeth, and acts as though he'd like to plant them in your leg. His eyes sparkle, his short tail lashes the ground like a flail, while he snorts like a porpoise. And this chap we took for a *pet !* Say, Doctor, do come down with your rifle ! We can't get near enough to kill him with a shot gun, and he has nearly gnawed that oak post through. Come quick, or we'll flee to the mountains. No more chucks for us—if you please !

---

THE EVER GLORIOUS.—It is right here. We hear the pop, bang, whiz that are the precursory symptoms of its arrival. The boys—from the oldest down to Fritz, have supplied themselves with the usual noise-making contrivances, and touch one off now and then just to see whether they are ready to afford their *quantum sufficit* of din and racket when the auspicious moment arrives. Other people's boys are wandering about the streets and back lanes, in search of barrels, boxes, hogsheads or any other combustible materials that may be found lying around loose, These are piled away in some mysterious nook to be brought out at the proper time to burn, blaze, and crackle their glory to the scene. The amount of "bang" in a boy cannot be estimated by his size—he'll fire a cannon if he can by any means reach its touch-hole. The small chinese cracker is a miserable squib ; he wants a "dolly varden"—"somethin' that'll crack loud," he says. He looks longingly aloft during a thunder storm and when your neighbor's chimney is knocked into a cocked hat by a thunder-bolt, his eyes dance with delight while he claps his hands and joyously exclaims—"wasn't that a peeler !"

We see it brewing—the symptoms point toward a regular pandemonium of disturbing sounds. We note the preparation—see the crackers of huge proportions, cannons of assorted sizes, and rockets rivaling your water cooler in bigness,—da, da—we go to Bodines, and if our house remains upon its foundations, we will return to it on the 5th.

---

BUGGY.—Things are buggy, not to say wormy, now-a-days. Every flower, every plant in your garden, has its vexatious little parasites. Humpbacked caterpillars perform their little trapeze acts above your head and swing themselves gracefully, back and forth, within an inch of your nose. Great, scrawly spiders hop dexterously across your path and escape to the nearest tree, from which they spin their unseen webs that smear your face with dew in your morning walks. Little green worms, drop from the leaves above your head, and vie with each other to see which of them can light upon your neck at the first trial, and then play hide and seek up and down your spinal column. Many legged worms go scampering about, when you pick your book from the arbor table, and, while reading, big black ants,—winged and wingless—straggle up one leg, and escape down the other, when you bring your hand upon the place where you supposed them to be. Microscopic flies dart into your eyes when you are riding, and nearly blind you with their acrid secretion. Flies light upon your ears and tickle your baldhead when at meals, while cockroaches creep into the jam. Midges, those little vexatious imps of Satan, thrust their needle pointed proboscides into your skin and drink your blood, unseen. While you write, all sorts of moths and bugs dart at your light and fall crippled and wingless upon your table, to scamper across the paper and dot your i's for you as they go. Mosquitos fly through your screened windows and sing their diabolical tunes in your ears by night, until you are weary with much brandishing of pillow or towel, when they pierce your quivering flesh with their bloodthirsty bills, then depart to the outer darkness, and sing their hallelujahs to the waiting hosts yet to come. Fat little wigglers roll themselves up in the solt wool of your carpets, and grow more plethoric still in your cayenne and scotch snuff.

Everything is buggy, grubby, or wormy,—confound it.

